# Health and Health Determinant Metrics for Cities: A Comparison of County and City-Level Data

**DOI:** 10.5888/pcd17.200125

**Published:** 2020-11-05

**Authors:** Ben R. Spoer, Justin M. Feldman, Miriam L. Gofine, Shoshanna E. Levine, Allegra R. Wilson, Samantha B. Breslin, Lorna E. Thorpe, Marc N. Gourevitch

**Affiliations:** 1 Department of Population Health, New York University School of Medicine, New York, New York

## Abstract

We evaluated whether using county-level data to characterize public health measures in cities biases the characterization of city populations. We compared 4 public health and sociodemographic measures in 447 US cities (percent of children living in poverty, percent of non-Hispanic Black population, age-adjusted cardiovascular disease mortality, life expectancy at birth) to the same measures calculated for counties that contain those cities. We found substantial and highly variable city–county differences within and across metrics, which suggests that use of county data to proxy city measures could hamper accurate allocation of public health resources and appreciation of the urgency of public health needs in specific locales.

**Summary**

**What is already known on this topic?**

Many local health departments develop city-level public health policies but lack city-level health data. This lack causes reliance on county-level data, which may misrepresent city populations.

**What is added by this report?**

We found substantial and highly variable city–county differences within and across 4 public health metrics, suggesting use of county-level data may mischaracterize health metrics in cities.

**What are the implications for public health practice?**

Use of county data to proxy city measures could hamper municipal public health policymaking. Public health officials concerned with cities should use city-level data whenever possible.

## Objective

When local officials develop and evaluate city health policies, they often cannot access city-specific health data, and instead rely on county data. Yet, a county’s characteristics may differ substantially from a city within it. Counties are usually larger than their cities, are mostly administered by different governmental entities, and contain overlapping, but distinct, populations, with different sociodemographic composition, health behaviors, and health conditions (1). As such, use of information from county-level data sources may skew data-driven efforts to allocate resources and target interventions (2,3).

## Methods

We evaluated the potential for bias by comparing estimates of 4 public health measures, calculated by using data from the City Health Dashboard (4) for 447 cities and their corresponding counties.

The City Health Dashboard (Dashboard) includes 497 US cities with a population of 66,000 or more, as of 2010, and to ensure all states were represented, 3 smaller cities (Burlington, Vermont; Cheyenne, Wyoming; and, Charleston, West Virginia) are included. The sample for the present analysis included the 447 Dashboard cities that are nested within their surrounding counties. Nested means that a minimum of 90% of the city population resides within the surrounding county, omitting the 53 cities that overlapped, or shared, county boundaries.

We then selected 4 measures to compare across city–county pairs: percent of non-Hispanic Black population, percent of children living in poverty, cardiovascular disease (CVD) deaths per 100,000 population, and life expectancy at birth. Percent of non-Hispanic Black (5) and percent of children living in poverty (6) were selected as demographic measures because of their consistent association with poor health outcomes. Percent of non-Hispanic Black is included as a demographic feature on the Dashboard for numerous Dashboard metrics.

For this analysis, percent of non-Hispanic Black and percent of children living in poverty were separately calculated at the city- and county-level by using the US Census American Community Survey, 2017 5-Year Estimates (7). We calculated CVD death rates using pooled, 2013–2015 restricted-use mortality data from the Centers for Disease Control and Prevention (CDC). We obtained city-level data through a data use agreement with CDC’s National Vital Statistics System (https://www.cdc.gov/nchs/nvss/index.htm) using our standing Dashboard agreement, and county-level data were obtained from CDC’s WONDER (Wide-ranging Online Data for Epidemiologic Research) website (https://wonder.cdc.gov/). We defined CVD deaths using a standard group of ICD-10 (International Classification of Disease, Revision 10) codes (8), with the following codes removed because their etiology differs substantially from that of more typical CVD mortality: I00–02 (acute rheumatic fever), I00–09 (chronic rheumatic heart diseases), I26–28 (pulmonary heart disease), and I30-I51 (other forms of heart disease) (9). CDC’s National Vital Statistics System, United States Small Area Life Expectancy Estimate Program (USALEEP) (https://www.cdc.gov/nchs/nvss/usaleep/usaleep.html) provides census tract-level, small area estimates of life expectancy at birth for the period 2010–2015. Using USALEEP, we produced aggregate city- and county-level life expectancy estimates by calculating tract population-weighted means. Life expectancy for 8 cities and 9 counties were not available from USALEEP.

Relative and absolute percent differences were calculated to establish whether observed differences in metric estimates were meaningful or not. Consistent with previous literature, absolute differences greater than 5% and relative differences greater than 15% were considered meaningful (10). Last, we calculated mean differences for city–county pairs for selected metrics and created a dot plot to visualize city-level differences in metrics.

## Results

Our analysis included 76,141,914 individuals residing in cities, representing 73.9% of the Dashboard population and 23.3% of the US population. In 91.5% of the analyzed cities, 100% of the city population resided in the containing county. The median percent of the county population living in the analyzed cities was 19.4% (interquartile range, 7.0%–42.3%). Paired *t*-tests for city–county differences across all 4 measures yielded *P* values less than 0.01 (Table). On average, the CVD mortality rate for a city was 30 per 100,000 population (16.7%) higher than in the containing county. Child poverty in cities exceeded that of the containing counties by an average of 2.65%. The percent of non-Hispanic Black residents was 2.5% higher in cities versus the containing counties. Life expectancy was also higher in cities by 0.18 years.

**Table:** Average Difference^a^ Between City and County Estimates for 4 Public Health Metrics in Large US Cities Completely Contained in their Counties (N = 447^b^)

Metric	Overall Metric Values		City–county Pair Comparisons			
	City, Mean (SD)	County, Mean (SD)	Mean Difference^c^ (SD)	Range	City–County Pairs with Meaningful Absolute Difference^d^ (> 5%)	City–County Pairs with Meaningful Relative Difference^e^ (> 15%)
Cardiovascular disease deaths (per 100,000)^f^	210.42 (58.79)	180.27 (34.47)	30.15 (46.68)	−39.10 to 268.80	380 (85.0%)	250 (55.9%)
Children in poverty, no. (%)^g^	23.49 (11.34)	20.84 (7.08)	2.65 (9.65)	−31.27 to 31.03	416 (93.0%)	342 (76.5%)
Non-Hispanic Black, no. (%)^g^	13.88 (15.51)	11.43 (10.96)	2.45 (10.43)	−35.17 to 56.30	422 (94.4%)	366 (81.9%)
Life expectancy, y^h^	78.95 (2.28)	78.77 (2.00)	0.18 (1.23)	−4.20 to 5.60	13 (2.9%)	7 (1.6%)

a Differences are calculated for city–county pairs by subtracting the county value from the city value.

b Cities of 66,000 or more as of 2010, wholly contained within their surrounding counties, plus 3 smaller cities to represent all states.

c Paired sample *t*-tests for all metrics were significant at *P* < 0.01.

d Absolute difference was calculated as the absolute value of the county value for a given metric subtracted from the city value, divided by the average of the city and county values and considered meaningful if the difference was > 5%.

e Relative difference was calculated as the absolute value of the county value for a given metric subtracted from the city value, divided by the county value and considered meaningful if the difference was > 15%.

f Analysis of city-level data cardiovascular disease death rates required restricted data, which were accessed through the National Vital Statistics System’s Research Data Center. The findings and conclusions in this paper are those of the authors and do not necessarily represent the views of the Research Data Center, the National Center for Health Statistics, or the Centers for Disease Control and Prevention.

g Children in Poverty and Percent Non-Hispanic Black were calculated using US Census, American Community Survey, 2017 5-year estimates (https://www.census.gov/programs-surveys/acs).

h Average life expectancy estimates were provided by the US Small Area Life Expectancy Estimation Program (https://www.cdc.gov/nchs/nvss/usaleep/usaleep.html).

We found substantial variations in city–-county differences: for all 4 metrics, the standard deviation of the city–county difference was larger than the mean of the difference. For example, poverty was on average 2.65% greater in cities than in the counties that contain them, but the standard deviation of the city–county difference for that measure was 9.65% (Table). Both absolute and relative differences were considered meaningful for the majority of city–county pairs for the CVD death rate, percent of non-Hispanic Black population, and percent of children in poverty (Figure).

**Figure:**
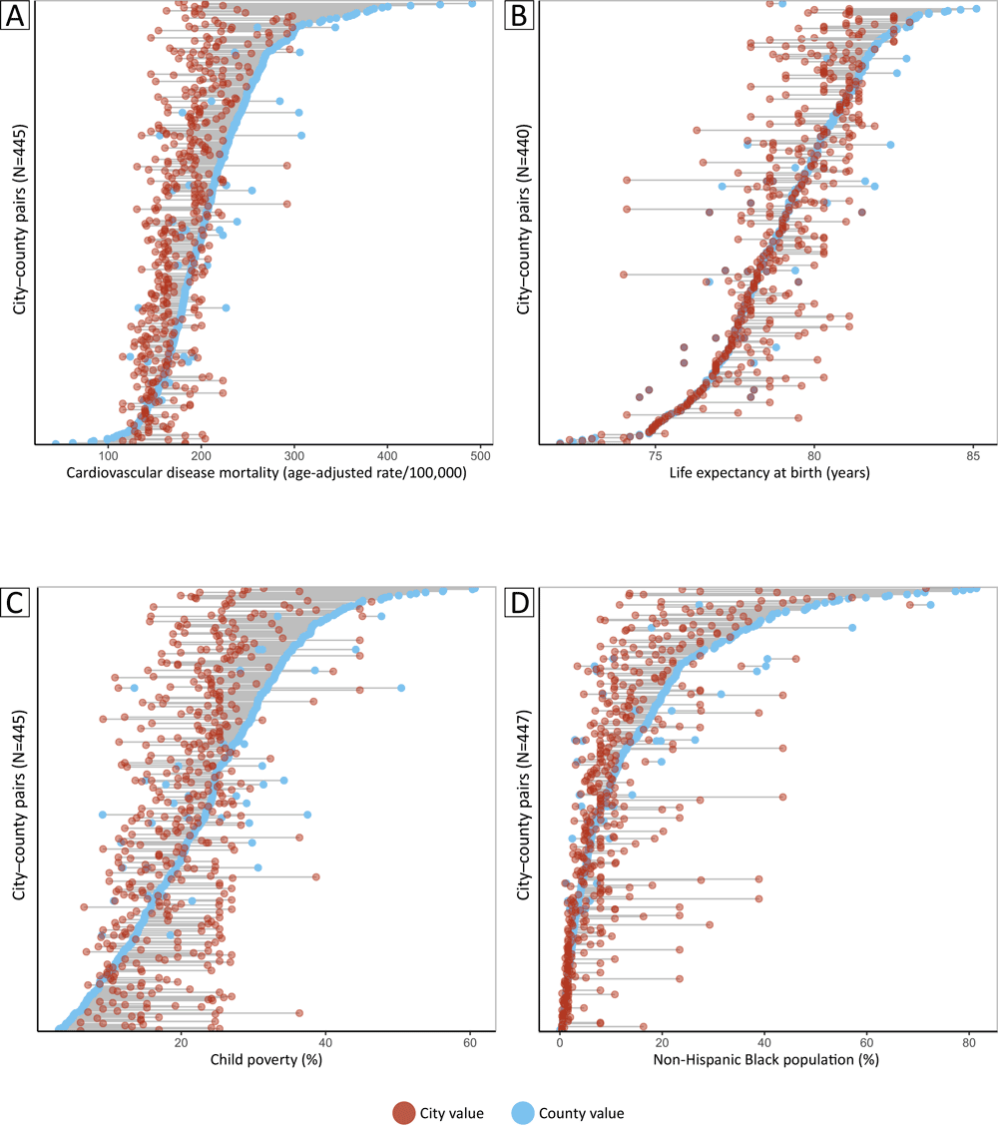
The dot plots display metric-level differences in city and county estimates for 447 large US cities that are completely contained by their surrounding counties. Data for some city–county pairs are missing on the y axis and were excluded from analysis. City–county differences vary greatly, both within and across metrics. A, Cardiovascular disease mortality; B, Life expectancy at birth; C, Child poverty; D, Non-Hispanic Black population.

## Discussion

Estimates for CVD mortality, the percent of non-Hispanic Black population, and the percent of children living in poverty were, on average, higher in cities than in counties, and most city–county pairs had meaningful absolute and relative differences for these 3 metrics. The higher percent of children living in poverty and non-Hispanic Black residents suggests that values of other health metrics may also be lower in cities than in counties that contain them, because both race and poverty are associated with health (11). However, life expectancy was longer, on average, in cities. This finding could reflect that life expectancy data incorporate some modeled estimates, recent increases in deaths of despair (deaths caused by suicide, accidental poisoning, and similar behaviors) outside of urban areas, or other reasons (12).

Although the general tendency was toward underrepresentation of poorer health outcomes when county-level metrics were compared with city-level metrics, observed differences varied substantially. As key public health actors, cities’ reliance on county-level estimates could in some cases attenuate the apparent need for action and resources. Although these findings do not include cities with populations less than 66,000, smaller cities typically include smaller proportions of the containing county’s population, so cities with lower population counts may be subject to similar or more substantial city–county differences.

Recent improvements in the ability of researchers to parse health-related data to specific geographic boundaries, and to generate modeled estimates when parsing is infeasible, have provided policy makers access to accurate city-level estimates, which were previously unavailable because of sparse data, methodologic limitations, or a lack of attention to city-level data. Such city-level data are increasingly publicly available from many sources, including the Dashboard. Cities’ use of granular city-level data should result in more accurately-targeted interventions and programs, which can contribute to better overall health outcomes.
